# RodentSQL: a software suite for colony management of animal protocols

**DOI:** 10.4155/fsoa-2018-0054

**Published:** 2018-07-27

**Authors:** Shrikant Pawar, Harshul Batra

**Affiliations:** 1 Department of Computer Science, Georgia State University, 34 Peachtree Street, Atlanta, GA, 30303, USA; 2 Department of Biology, Georgia State University, 34 Peachtree Street, Atlanta, GA, 30303, USA; 3 Neuroscience Institute & Center for Behavioral Neuroscience, Georgia State University, 789 Petit Science Center Atlanta, GA, 30303, USA

**Keywords:** colony management, recordkeeping, rodent, SQL database, transgenic

## Abstract

**Aim:**

Animal experiments tend to generate large-scale data. Some existing tools like Microsoft Excel and colony management cloud-based tools are either cumbersome or expensive. There is a need for an inexpensive and uncomplicated colony management software with specific individual database solutions.

**Methods:**

We have developed a database application named RodentSQL to meet most of the requirements of such animal colony management programs.

**Results:**

We present a user-friendly secure system for managing records of phenotype, genotype and metadata for comprehensive data analysis and mining. RodentSQL can be altered based upon user needs. We have successfully tested its usage in beta users for managing data of hamsters and mice.

**Conclusion:**

A central facility can share and benefit from this colony management system. RodentSQL can increase workflow efficiency and data security leading to significant cost savings and enhanced scientific results. RodentSQL is offered to the scientific community as an open source software.

## Lay abstract

Animal experiments tend to generate large-scale data. Some existing tools like Microsoft^®^ Excel (WA, USA) and colony management cloud-based tools are either cumbersome or expensive. Addressing this concern, we have developed a database application named RodentSQL to meet most of the requirements of such animal colony management programs, which can lead to significant cost savings and enhanced scientific results.

Animal models are a critical part of biomedical research. Their genetic and physiological similarity to humans and experimental tractability are a few of the important attributes accounting for their increasing usability in research. Russell and Burch outlined the three ‘Rs’: replace, reduce and refine [1,2] for the ethical use of animal models in research. Data integration and management is a significant issue in all animal experiments and appropriate bioinformatics support is a necessity. Despite the importance of data management, handwritten laboratory notebooks or spreadsheets are used for managing animal colonies. Simple adoption makes such *ad hoc* data management approaches useful, but errors due to scale-up issues limit their usage. Multiple-user accessibility issues with spreadsheets and cumbersome paper trails for paper records further limit their usability for metadata management studies. Also, such methodologies lack data-mining, analysis and real-time reminder capabilities which are necessary for effective and error-free rodent colony management. Spreadsheets, in particular, are not well-suited for managing the complex data structures (such as breeding schemes) required for effective scientific data and colony management.

Proper and secure storage of data in a central database seems a priority. Data of various types like standard operating procedures, experimental and housing conditions need appropriate storage separations. Furthermore3, redundant information for storage and retrieval needs to be reduced for saving resources. A central data resource would be essential to access all the information rather than multiple distributed spreadsheet files. In the main, animal lines from all over the world are imported for primary phenotyping and bred for secondary or tertiary phenotyping, which needs shared resources like rooms, racks, cages, sex, genotype, date of birth, origin, date/reason of death and genetic modifications tracking information. There have been attempts in past to develop such management software [3–7] and some of the currently available commercial tools are listed in Table 1.

**Table 1.** **Some of the currently available tools are listed.**

**Database**	**Charge**	**Database**	**Operating system**	**Website**

MausDB	Free	Oracle	No info	www.nervenet.org/

JCMS	Free	MySQL	Windows, Mac	http://colonymanagement.jax.org/

mLims	No info	No info	No info	https://bioinforx.com

Facility	No info	No info	No info	www.locustechnology.com/

SoftmouseDB	Paid	Cloud	Windows, Mac	https://softmouse.net/

MouseJ	Free	Windows. NET	Windows	http://mousej.org/

Mouse colony	Paid		Windows, Mac	http://mousecolony.com/Home.html

PyRAT	Paid	MySQL	Windows, Mac	www.scionics.com/pyrat.html

ezColony	Paid	Cloud	Windows, Mac	https://ezcolony.com/

Mosaic vivarium	Paid	No info	No info	https://mosaicvivarium.net/

Presented here, RodentSQL is a freely available and supported research tool that can be configured for a single user or multiple users. The target audience includes animal colony managers, lab supervisors and animal breeders. The application provides experimental workflows and data tracking by allowing real-time access to the database from any networked computer.

## Methods & design

### Workflow management for software

**Figure 1.**
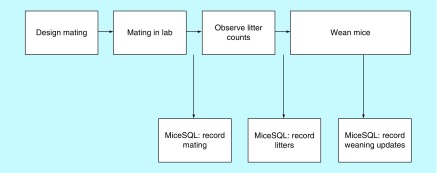
**Depiction of the workflow management for software.**

There is a straightforward workflow involved with the software. Upon installation, the user gets a window to login to the system with its unique username and password. The same username and password connect the user to his SQL database space. Once logged in, the user can start by creating the new project by entering a unique ID and respective information for the owner, such as number of male and females, mouse lines, strains, gene information, if any, date received, genotype, physical, and cage tags by recording it in the database. Each of the records, updates and deleted information gives a validation screen for the user to verify the change. The user can then move ahead to the following tabs for entering additional information. Unique ID would be the important number for all these tabs as all the information entered would be associated with this field. Figure 1 depicts the workflow management for software.

### Implementation

RodentSQL has been developed on a Mac OS X operating system using MySQL as the relational database management system and Java as the scripting language. All the fully functional software parts and packages can be installed on a Mac OS X operating system in less than 15 min. The hardware requirements of RodentSQL on the server side are moderate. The server we utilized is GSU Orion with CentOS 6.7 64-bit, 6x IBM System x3850 x5, Intel Xeon Processor E7–4850, 4 CPUs (10 cores per CPU), 2.0 GHz processors, 512 GB RAM and 2 TB of scratch storage for jobs. Compilation of all the java codes was conducted on NetBeans integrated development environment, and all the java classes and compilation codes are submitted on a GitHub repository, which can be found on the corresponding authors account [8]. The database has 11 columns of text field table that are updated interactively by the user. Furthermore, information upload is standardized as specific parameters such as the date have to be inputted in a distinct format; this helps in the retrieval process. Checking of data type (oat, integer, text and Boolean), mouse IDs and dates are essential before input into a database. Errors with dates, or invalid parameters or wrong data types, cause a halt in a workflow. Bounds and ranges have also been considered and need to be used according to the user instructions.

### Security

We have implemented strong security measures for authentication of the SQL server. Kerberos protocol uses a number of encrypted messages to authenticate SQL server and the passwords are not passed across the network. Authentication is more reliable and managing it can be reduced by leveraging active directory groups for role-based access to SQL server. The sysadmin (sa) account is vulnerable when it exits unchanged so we have disabled the sa account on the SQL server instance. We chose to give options for complex passwords for sa and all other SQL-server-specific logins on SQL server and checked in the ‘Enforce password expiration’ and ‘Enforce Password policy’ options for sa. We haven't allowed to explicit grant control server permission because logins with this permission get full administrative privileges. For permissions to users, a built-in _xed server roles and database roles or creating own custom server roles and database roles can be achieved. Guest user exists in every user and system database, which is a potential security risk in a lockdown environment because it allows database access to logins who don't have associated users in the database. We have restricted this access and also accesses to user and system stored procedures. Furthermore, we have used common specific TCP ports (excluding 1433 and 1434) instead of dynamic ports. SQL server browser service is only running on SQL servers and secure SQL server error logs and registry keys using NTFS permissions are utilized as they can provide a great deal of information about the SQL server instance and installation.

### Individual IDs

The RodentSQL application is composed of colony management, experiment/protocol management and report functions. The central entity of the RodentSQL module is the mouse id. Every other entity has a relationship with the mouse ID which facilitates complete tracking over other parameters like generations, genotype tracking and mouse caging. Individual mouse IDs are essential to track the records. It is a unique ID that can get you all of the information associated with a specific record. We built such a structure to ease information retrieval.

### Genealogy

**Figure 2.**
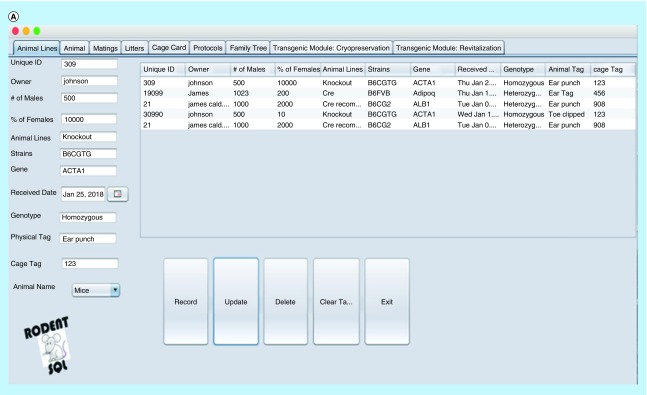

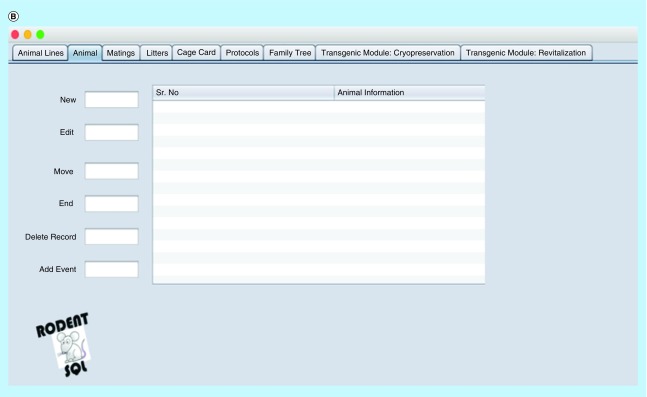

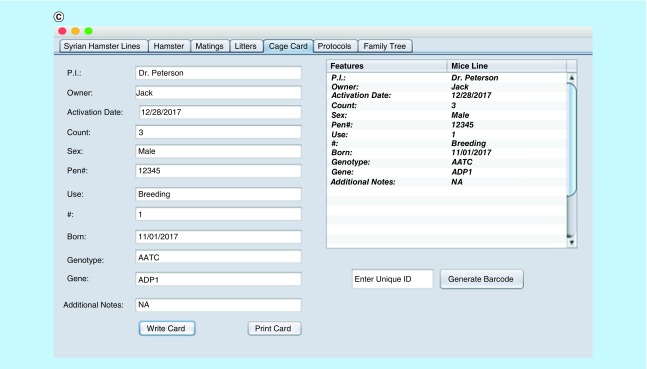

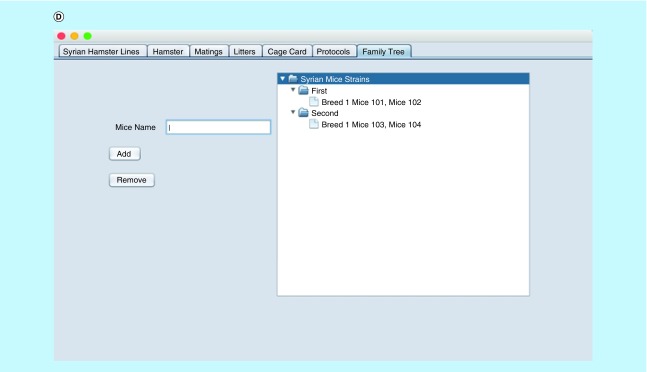
**The snapshot of some software interfaces.** **(A)** Interface window for recording, updating and deleting animal records. **(B)** Cage card writing window. **(C)** Pedigree/family tree window. **(D)** Printing cage card and generating a unique bar code for cage cards.

The RodentSQL pedigree/family tree tracking tool connects the database and graphically displays pedigree trees (Figure 2). It allows drawing of an ancestor or progeny tree using the selected mouse. This technique is useful for tracking generations of mice and the weaning process.

### Cage cards

RodentSQL allows users to print cage cards with information from the database about the mice from the cage (Figure 2). We have provided a unique bar code associated with each cage which can be printed and allows users to track information from the cage back to the database. Cage cards consist of investigator, activation date, mice count, sex, born, genotype, gene and mating information.

### Transgenic module

Cryopreservation: we have incorporated a unique ID for the cage tag, with sire and dam information and methods of preservation (super ovulation, hormone priming, IVF, simple sperm cryopreservation and others), along with tank temperature, location, number and the type of embryo information. Revitalization: we have incorporated a unique ID for cage tag, thaw number, revitalization number, reservation via transfer and founder's information. User can track back the transgenic module cryopreservation information from this tab with its unique cage tag

## Results & discussion

RodentSQL is built on a Mac OS X operating system, MySQL database with Java as a programing language. We have used a nonredundant storage ensuring integrity and consistency of data. A central database with an improved backup strategy has been implemented to prevent hack and data loss. The largest number of colonies that may be managed by RodentSQL is 1000 colonies. This number varies; for the beta users, 1000 colonies were tested, but this number can go up and is not limited to 5000 or more for single users. Due to limitations of server space, we cannot assure more than 5000 colonies/single users at this stage, although the upgrade will be assimilated in our subsequent versions of this software. The design is adaptable to other experimental models like aquatic animals and fruit flies, if needed. RodentSQL is intuitive, thereby requiring minimal user training. Simple user interface with tabs separating each function is included. Animal lines, animal information, mating, litters, cage cards, protocols and family are the broad section tabs for each project. Data in each tab can be recorded, updated and deleted with respective buttons from the SQL database. Figure 2 shows the snapshot for each of these interfaces. It can be easily installed and customized. From testing RodentSQL on a beta user, we found that it helps to reduce the amount of time spent with mouse colony management. A central facility can share the mouse space and can benefit from this mouse colony management system. While distributed spreadsheet files or laboratory journals seems cumbersome, our solution manages such data cleanly.

Availability and requirements: Project name: RodentSQL. Operating system: platform-independent. Programming language: Java. Other requirements: server: Apache 1.3 or above, MySQL 4.23 or above; client: OS: Mac OS X. License: GNU General Public License. Any restrictions for use by nonacademics: none. Known issues and test notes will be published with each software release version. Available from [8].

## Conclusion & future perspective

RodentSQL is used to meet the functional requirements of an experimenter working with mice lines to ease the record tracking puzzlement. User acceptance usually comes from usefulness and usability of the tool. Taking issues with already available software into consideration we attempted to develop a user-friendly interface with convenience, and more features such transgenic mode and ease of use. Some of the advantages of RodentSQL are listed in Table 2. RodentSQL can increase both workflow efficiency and data security and thus produce significant cost savings and enhanced scientific results. The application was designed with extensive input from end users and addition of new features including support for additional model organisms, enhanced vivaria management functions, and support for associating samples with specific experiments will be considered. This software has the potential to go through many additions in future development. Some of them include integration of tools for basic statistical analysis, data visualization, integration of ontologies and controlled vocabularies for the collection of phenotype data.

**Table 2.** **Advantages of RodentSQL over commercially available colony management software.**

**Model type**	**Cost**	**Usability**	**Protocols**	**Lab size**	**Animal models**	**Transgenic module**

RodentSQL	+	+	Yes	++	All Rodents	No

MausDB	+	+	No	+	Mouse	No

JCMS	++	+	Yes	++	Mouse	No

mLIMS	+++	++	No	+	Mouse	No

SoftmouseDB	+	+	Yes	+++	Mouse	No

MouseJ	++	++	No	+	Mouse	No

Mouse colony	+++	+	No	+	Mouse	No

PyRAT	++	++	Yes	+++	Rat	Yes

ezColony	+	+	Yes	++	Mouse	No

**Practice points**

- Data of various types such as standard operating procedures, experimental and housing conditions needs appropriate storage separations.
- RodentSQL is a freely available and supported research tool. It can be configured for a single user or multiple users. The target audience includes animal colony managers, laboratory supervisors and animal breeders.
- The application provides experimental workflows and data tracking by allowing real-time access to the database from any networked computer.
- We have used a nonredundant storage ensuring integrity and consistency of data. A central database with an improved backup strategy has been implemented to prevent hacking and data loss.
- The design is adaptable to other experimental models like aquatic animals and fruit flies if needed.
- RodentSQL is intuitive, thereby requiring minimal user training. Simple user interface with tabs separating each function is included.
- RodentSQL can increase both workflow efficiency and data security and thus produce significant cost savings and enhanced scientific results.
