# Addendum

**DOI:** 10.2144/fsoa-2018-0054c1

**Published:** 2019-10-03

It has come to our attention that the Short Communication by Shrikant Pawar & Harshul Batra, ‘RodentSQL: a software suite for colony management of animal protocols’, which appeared in Volume 4, Issue 8 of *Future Science OA* (4[8], FSO330 [2018]), was published erroneously as a duplicate publication elsewhere, and subsequently retracted. This notification serves to assure our readers that this version of the article, published in *Future Science OA*, is the correct version.

